# Transcriptional regulation of central carbon metabolism in *Pseudomonas aeruginosa*

**DOI:** 10.1111/1751-7915.13423

**Published:** 2019-06-11

**Authors:** Stephen K. Dolan, Greicy Pereira, Rafael Silva‐Rocha, Martin Welch

**Affiliations:** ^1^ Department of Biochemistry University of Cambridge Cambridge UK; ^2^ Faculdade de Medicina de Ribeirão Preto Universidade de São Paulo Ribeirão Preto Brazil

## Abstract

Microbes such as *Pseudomonas aeruginosa* are often challenged by rapidly changing nutritional environments. In order to adapt to these shifts in nutrient availability, bacteria exert tight transcriptional control over the enzymes of central metabolism. This transcriptional control is orchestrated by a series of transcriptional repressors and activators. Although a number of these transcription factors have been identified, many others remain uncharacterized. Here, we present a simple pipeline to uncover and validate the targets of uncharacterized transcriptional regulators in *P. aeruginosa*. We use this approach to identify and confirm that an orthologue of the *Pseudomonas fluorescens* transcriptional regulator (RccR) binds to the upstream region of isocitrate lyase (*aceA*) in *P. aeruginosa*, thereby repressing flux through the glyoxylate shunt during growth on non‐C2 carbon sources.

## Graphical abstract

Transcriptional control in bacteria is orchestrated by a series of transcriptional repressors and activators. Although a number of these transcription factors have been identified, many others remain uncharacterized. We have developed a robust pipeline to identify and functionally annotate new transcriptional regulators in *Pseudomonas aeruginosa*. We use this approach to identify and confirm that an orthologue of the *Pseudomonas fluorescens* transcriptional regulator (RccR) binds to the upstream region of isocitrate lyase (*aceA*) in *P. aeruginosa*, thereby repressing flux through the glyoxylate shunt during growth on non‐C2 carbon sources.

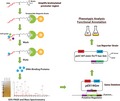

## Introduction

*Pseudomonas aeruginosa* (PA) is a metabolically versatile, Gram‐negative opportunistic pathogen capable of causing chronic lung infections in patients with cystic fibrosis (CF). The remarkable metabolic plasticity of the organism can be attributed to its large repertoire of encoded regulatory proteins – around 9% of the PA genome is dedicated to transcriptional regulation (Stover *et al*., 2000; Meadows and Wargo, 2018).

When bacteria grow in the presence of preferred carbon sources, alternative carbon assimilation pathways are often transcriptionally repressed (Rojo, 2010). However, and despite decades of research into bacterial central metabolism, the transcriptional regulation of numerous key metabolic nodes in *Pseudomonas aeruginosa* remains uncharacterized (Galán‐Vásquez *et al*., 2011). For example, the glyoxylate shunt (GS) is an anaplerotic pathway which facilitates microbial growth on acetate as a sole carbon source (Crousilles *et al*., 2018). The presence of an intact GS is essential for infection by several prominent microbial pathogens, including *Candida albicans*,* Mycobacterium tuberculosis* and *Pseudomonas aeruginosa* (Dolan and Welch, 2018). The GS is also important for butanol assimilation in *P. putida* (del Cuenca *et al*., 2016). The *P. aeruginosa* GS genes *aceA* (encoding *iso*citrate lyase, ICL) and *glcB* (encoding malate synthase G, MS) are known to be highly transcribed when the organism is grown on acetate as a sole carbon source, but are tightly repressed during growth on glycolytic carbon sources, such as glucose (Crousilles *et al*., 2018).

The affinity of transcriptional regulators for their target operator DNA may be dependent on the presence of metabolic cues (ligands) which modify the structure of the regulator. This allows even subtle changes in the cellular environment to elicit rapid transcriptional responses (Bateman, 1999). Crucially, this does not require a change in net expression of the regulator. This limits the applicability of comparative ‘omics in elucidating the identity of these regulators, so alternative approaches need to be developed/refined. Here, we present a rapid pipeline to uncover and validate the targets of uncharacterized transcriptional regulators in *P. aeruginosa* (and potentially, also other pseudomonads). Using this method, we uncover a transcriptional regulator which represses the GS in *P. aeruginosa* during growth on non‐C2 carbon sources, allowing for the rapid and reversible transcriptional activation of this key pathway in response to changes in carbon skeleton availability.

## Results and discussion

### Workflow

**Figure 1:**
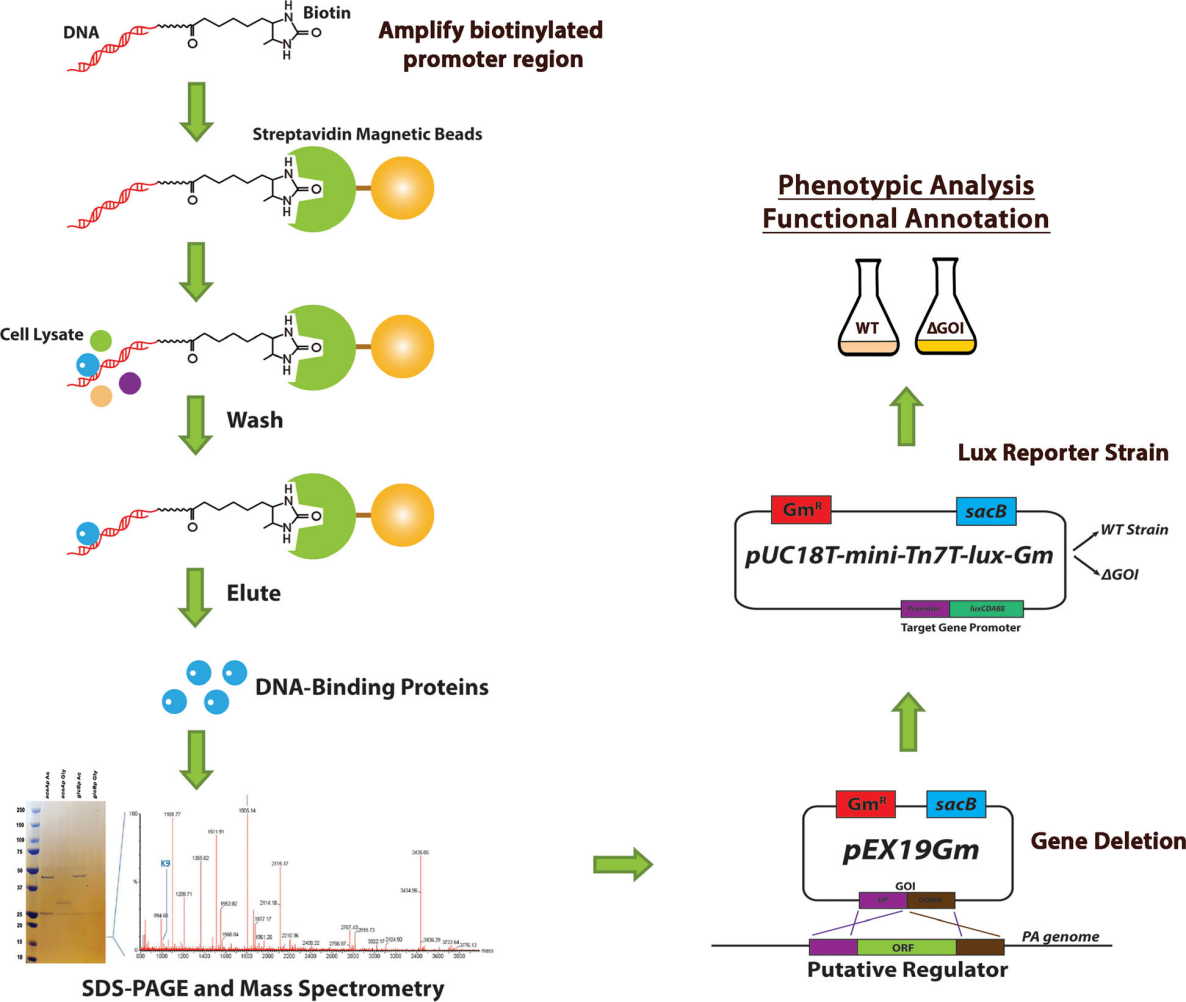
Schematic of the workflow pipeline.

The workflow for this pipeline is illustrated in Fig. 1.

- First, culture conditions where transcription of the gene of interest (GOI) is maximally activated or repressed (‘ON’/’OFF’ conditions, identified from ‘omic data, etc.) are confirmed using a plasmid‐borne promoter:luciferase reporter assay (Fig. 2).
  **Figure 2:**
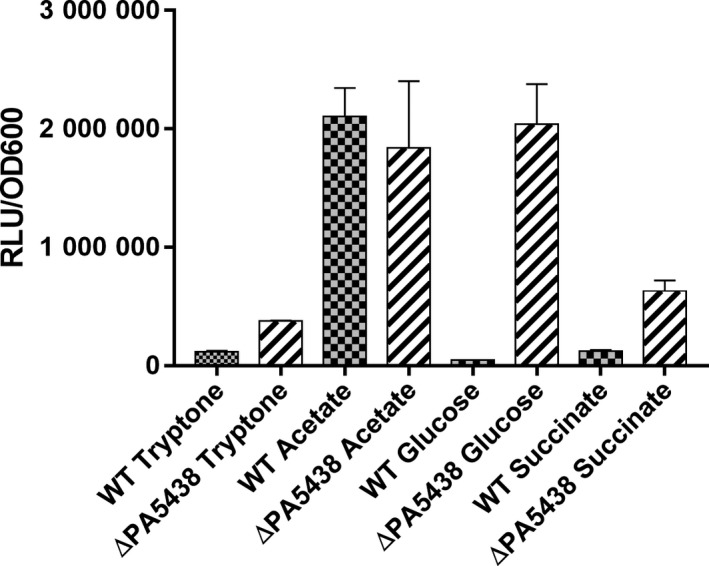
*aceA*:lux expression for the wild‐type and the Δ*PA5438* mutant grown in MOPS minimal medium containing tryptone, acetate, glucose or succinate, as indicated. Gene expression was measured as relative light units (RLU) derived from the activity of the expressed *lux* enzymes. RLU values are normalized to the culture OD_600_. Data represent mean ± SD from three biological replicates.
- The region upstream of the GOI (incorporating the promoter and associated operator sites) is PCR‐amplified using a non‐modified forward primer and a biotinylated reverse primer. This amplified segment of DNA is then bound at high immobilization density onto streptavidin‐coated magnetic beads (Dynabeads). In parallel, cells from cultures grown in the ‘max ON’ and ‘max OFF’ conditions identified in step 1 are harvested, washed and lysed to generate concentrated protein lysates.
***Key point**: for best results, it is important to maximize the density of immobilized DNA fragment on the beads*.
- The immobilized upstream region of the GOI is used to capture protein(s) from the concentrated cell lysates in step 2. The beads are washed to remove non‐specifically bound proteins, and the DNA‐bound regulators are released by treatment with a high‐salt buffer (containing 1M NaCl).
***Key point**: transcription factors are often present at very low concentration in the cell, so the lysate should contain a high concentration of protein*.
***Key point**: best results were obtained when the pulldowns were carried out in the presence of non‐specific competitor DNA (we used salmon sperm DNA,* Fig. S1 *)*.
- The eluted proteins are concentrated by acetone precipitation and then analysed by SDS‐PAGE (we routinely use 4–20% polyacrylamide gradient gels for these purposes). Following staining to reveal the resolved proteins, bands of interest are excised and sent for MS‐MS fingerprinting.
- The identified transcriptional regulator(s) are then deleted from the *P. aeruginosa* genome using established methods, and the promoter:luciferase reporter from step 1 is then introduced into the mutant(s) enabling comparison of the transcriptional profile of the GOI in the deletion mutant with the wild‐type progenitor.
- The pipeline above, which fuses biochemical and genetic approaches, can be readily extended to include, for example, site‐directed mutagenesis of the regulator itself, or of its binding site on the DNA. The use of stable, chromosomally integrated *lux* reporter constructs means that the method is also tolerant of plasmid‐based complementation, if required.

### Advantages of the method

Our approach is rapid, easy, inexpensive and internally validated. Due to solubility and expression issues, bacterial transcription factors are notoriously difficult to overexpress in soluble form and purify for characterization *in vitro*; crucially, our approach does not rely on this. Furthermore, the method is easily adapted to multiplexing, enabling several DNA‐binding sites to be probed simultaneously.

### Example – regulation of the glyoxylate shunt enzymes in *P. aeruginosa*

The detailed experimental procedure is described in Supplementary Information.

The genes encoding the GS enzymes, isocitrate lyase and malate synthase (*aceA* and *glcB*, respectively) have been shown previously to be transcriptionally activated during growth on acetate and repressed during growth on glycolytic carbon sources (Diaz‐Perez *et al*., 2007; Ha *et al*., 2018).

To investigate the regulation of the GS genes further, we first cloned the upstream region of *aceA* and *glcB* separately into the *lux* reporter construct, pUC18T‐mini‐Tn7T‐lux‐Gm. This construct integrates in single copy into a neutral site in the chromosome, enabling a convenient readout of the target promoter activity without the complications arising from gene copy number effects associated with plasmid‐borne reporters. We used these constructs to confirm and quantify the transcriptional activity driven by the upstream regions of *aceA* and *glcB* during the growth on acetate, glucose, succinate or tryptone as sole carbon sources (the data are shown in Fig. 2 for *aceA* and Fig. S5 for *glcB*). These data confirm that the transcription of these genes is strongly stimulated during growth on acetate, but remains low in glucose, succinate or tryptone.

The *glcB* and *aceA* upstream regions (ca. 300 and 600 bp in length, respectively; the aceA gene is preceded by one of the longest intergenic regions in the PAO1 chromosome) were PCR‐amplified, incorporating a biotin tag on the 3′ end. These amplicons were then attached to magnetic streptavidin‐coated Dynabeads. We found that it was crucially important to ensure that the streptavidin sites on the beads were saturated with biotinylated probe. The beads were then mixed with freshly prepared protein lysates obtained from *P. aeruginosa* grown on glucose or acetate as a sole carbon source. Following 30‐min incubation at room temperature, the beads were washed, and the bound putative regulator(s) were eluted using a high‐salt buffer. The eluted proteins were concentrated and analysed by SDS‐PAGE. A protein (approx. 30 kDa in mass) from lysates obtained from cells grown on glucose, but not from cells grown on acetate, was found to bind to the *aceA* upstream region (Fig. S1). MS/MS fingerprinting of the corresponding protein band revealed it to be PA5438, a probable transcription factor of the RpiR family.

**Figure 3:**
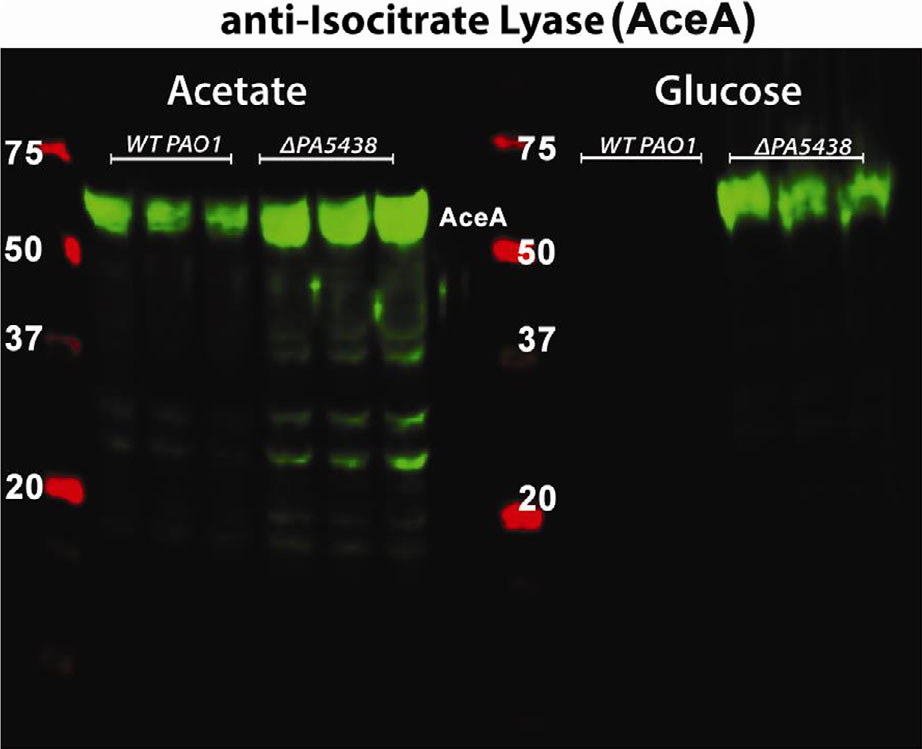
Western blot to detect isocitrate lyase (AceA, 58 kDa) expression in wild‐type *P. aeruginosa* (strain PAO1) and in an otherwise isogenic Δ*PA5438* mutant during growth on acetate and glucose (as indicated) sole carbon sources. Molecular mass markers in kDa (red) are indicated. Note how AceA expression is repressed in the wild type during growth in MOPS‐glucose, but not in the Δ*PA5438* mutant. Each lane represents an independent biological replicate.

RpiR‐family transcription factors are widespread in bacteria and are known to regulate sugar catabolism, such as maltose, glucose and ribose metabolism and the pentose phosphate pathway. RpiR‐family regulators can act as either activators or repressors of transcription and are composed of a characteristic N‐terminal DNA‐binding helix–turn–helix (HTH) domain and a C‐terminal sugar phosphate binding domain (Sørensen and Hove‐Jensen, 1996; Bateman, 1999). To investigate the role(s) of PA5438 further, we deleted PA5438 from PAO1, as described in the *Supplementary Information*. The *aceA* and *glcB* lux reporter constructs were introduced into the resulting ΔPA5438 mutant, allowing us to test whether PA5438 impacts on expression of these target genes. Both target genes showed greatly increased transcription during growth in non‐C2 carbon sources, indicating that PA5438 normally represses gene expression on these carbon sources. The control promoter (*cco1*) showed comparable expression levels in the ΔPA5438 mutant and the PAO1 wild‐type (Fig. S6), suggesting that this mutant specifically affects the glyoxylate shunt genes. In addition, we also used Western blotting of cell lysates from the wild‐type and ΔPA5438 mutant to investigate the impact of PA5438 deletion on ICL levels during growth on glucose versus acetate (Fig. 3). Unlike the situation in the wild type, ICL levels remained high during growth of the ΔPA5438 mutant on glucose. ICL expression was also de‐repressed in the ΔPA5438 mutant during growth on succinate or tryptone (Fig. S7). These data indicate that the transcriptional de‐repression observed in the ΔPA5438 mutant feeds through to the protein level. Similarly, we also used Western analyses to investigate whether malate synthase (MS) was de‐repressed during growth on non‐C2 substrates. It is shown in Fig. S5. This was an interesting result because *aceA* (encoding ICL) and *glcB* (encoding MS) are unlinked in *P. aeruginosa*, suggesting that PA5438 may act independently to repress both genes. By contrast, in *E. coli* and other enterics, ICL (*aceA*) and MS (denoted *aceB* in *E. coli*) are encoded in a single operon.

Interestingly, the ΔPA5438 mutant grew at a rate comparable with that of PAO1 on acetate, glucose, succinate or tryptone (Fig. S3). This suggests that (over the short term) inappropriate expression of GS enzymes does not impose an unmanageable selective pressure on *P. aeruginosa*. One reason for this may be that once the enzymes have been synthesized, they are still subject to stringent control mechanisms based on allostery and reversible covalent modification (Crousilles *et al*., 2018). PA5438 is 88% identical to the recently identified *P. fluorescens* GLX regulator, RccR, which binds KDPG (Campilongo *et al*., 2017). In *P. fluorescens*, RccR has been shown to bind to a consensus motif (ATGTAG‐X_14_‐CACTACAT) located upstream of its target ORF(s). A similar sequence motif (GTGTAG‐X_14_‐CACTACAA) is centred 447 nucleotides upstream of the *aceA* translational start site and around 55 nucleotides upstream of the transcriptional start site. Subsequent truncation analyses of the long upstream region of *aceA* confirmed that this segment of DNA was essential for both acetate inducibility and transcription *per se* (*data not shown*).

Current efforts are aimed at characterizing a suite of novel transcriptional regulators captured using pulldowns with the upstream regions of other genes of interest.

## Conflict of interest

The authors confirm that they have no conflict of interest.

## Supporting information


**Appendix S1.** Supplementary Materials and Methods.
**Table S1.** Oligonucleotide primers used in the study.
**Table S2:** Bacterial strains and plasmids used in this study.
**Fig. S1.** Capture of putative regulatory proteins on immobilized DNA.
**Fig. S2.** Mass spectrometric identification of protein bands (A‐C) which bound to the *aceA*/*glcB* promoter regions.
**Fig. S3.** Growth curves of *P. aeruginosa* PAO1 wild‐type (red) compared with the *ΔPA5348* mutant (blue) grown on various carbon sources.
**Fig. S4.** Quantitation of *aceA* transcriptional activity in wild‐type *P. aeruginosa* (PAO1, blue line) compared with *aceA* transcription in a *ΔPA5348* mutant (red line).
**Fig. S5.** *glcB*:lux expression for the wild‐type and the Δ*PA5438* mutant grown in MOPS minimal medium containing tryptone, acetate, glucose or succinate, as indicated.
**Fig. S6.** *cco1*:lux expression for the wild‐type and the Δ*PA5438* mutant grown in MOPS minimal medium containing tryptone, acetate, glucose or succinate, as indicated.
**Fig. S7.** Western blot showing deregulated expression of AceA (59 kDa) and GlcB (79 kDa) in a *ΔPA5348* mutant compared with the wild‐type during growth on the indicated carbon sources.
