# Supplementary material for: Transcriptional regulation of central carbon metabolism in Pseudomonas aeruginosa
Source: Microb Biotechnol. 2019 Jun 11;13(1):285–9. doi: 10.1111/1751-7915.13423 (PMC6922535; doi:10.1111/1751-7915.13423)
Supplement: Supplementary file 1 — Appendix S1. Supplementary Materials and Methods. Table S1. Oligonucleotide primers used in the study. Table S2: Bacterial strains and plasmids used in this study. Fig. S1. Capture of putative regulatory proteins on immobilized DNA. Fig. S2. Mass spectrometric identification of protein bands (A‐C) which bound to the aceA/glcB promoter regions. Fig. S3. Growth curves of P. aeruginosa PAO1 wild‐type (red) compared with the ΔPA5348 mutant (blue) grown on various carbon sources. Fig. S4. Quantitation of aceA transcriptional activity in wild‐type P. aeruginosa (PAO1, blue line) compared with aceA transcription in a ΔPA5348 mutant (red line). Fig. S5. glcB:lux expression for the wild‐type and the ΔPA5438 mutant grown in MOPS minimal medium containing tryptone, acetate, glucose or succinate, as indicated. Fig. S6. cco1:lux expression for the wild‐type and the ΔPA5438 mutant grown in MOPS minimal medium containing tryptone, acetate, glucose or succinate, as indicated. Fig. S7. Western blot showing deregulated expression of AceA (59 kDa) and GlcB (79 kDa) in a ΔPA5348 mutant compared with the wild‐type during growth on the indicated carbon sources. [file MBT2-13-285-s001.docx]

**Supplementary Information**

**Media and growth conditions**

*P. aeruginosa* strain PAO1 (Holloway et al., 1979, 1994) was routinely grown in lysogeny broth (LB; comprising 1% tryptone, 1% NaCl, and 0.5% yeast extract) at 37°C with good aeration (rotary shaking at 250 rpm). Overnight liquid pre-cultures served as the inocula for each experiment. The strains and plasmids used in this study are listed in Table S2. The overnight pre-cultures for biological replicates were started from separate clonal source colonies on streaked LB-agar plates. Strains were cultured in MOPS (3-(N-Morpholino)-propanesulfonic acid) media with the relevant carbon sources (1% (w/v) tryptone, 15mM glucose, 40mM acetate, or 20mM succinate) (LaBauve and Wargo, 2012). Cell growth was monitored as optical density at a wavelength of 600 nm (BMG Labtech FLUOstar Omega microplate reader).

**Construction of an in-frame PA5438 deletion mutant in *P. aeruginosa* PAO1**

Flanking regions (800-1000 bp) upstream and downstream of PA5438 were PCR amplified using primers 1 and 2, and 3 and 4, respectively (Table S1). Upstream and downstream regions were then overlapped and cloned into the suicide vector pEX19Gm using Gibson assembly as described previously (Husang & Wilks, 2017). The resulting plasmid was then introduced into *P. aeruginosa* by electroporation and transformants were selected on LB plates containing 50 µg/ml gentamicin. Putative deletion mutants were selected *via* *sacB*-mediated counter-selection on 15% sucrose-containing plates and confirmed by PCR.

**Generation of luciferase-promoter fusions**

Translational reporters were made by fusing the upstream sequence of the gene of interest (GOI) with *luxCDABE* using primers 5 and 6 (*aceA* upstream region), 7 and 8 (*glcB* upstream region) or 9 and 10 (*coo1* upstream region) in Table S1. The respective primers incorporated a BamHI site to the 5’ ends of the DNA segment and an XhoI site to the 3’ ends of the DNA segment. Purified PCR products were digested with BamHI/XhoI and ligated to similarly-digested pUC18T-mini-Tn7T-lux-Gm plasmid (Choi and Schweizer, 2006). Each mini-Tn7-lux element was integrated, separately, into the chromosome of PAO1 (at a highly conserved site downstream of the *glmS* gene). This was done by co-electroporation of the respective reporter construct along with the helper plasmid pTNS2, as previously described (Heath Damron et al., 2013). Transconjugants were selected on LB plates containing 50 μg/ml gentamicin. The genotype of each *lux* insertion derivative was confirmed by PCR.

**Luciferase-promoter assay**

Luciferase activity and OD_600_ readings were measured using a BMG Labtech FLUOstar Omega microplate reader. Strains were cultured in MOPS minimal media containing the indicated carbon source (100 µl culture volume) in 96-well microplates (Greiner bio-one, F-Bottom, Black), sealed with gas-permeable imaging seals (4titude - 4ti-0516/96). Luciferase expression was assessed every 30 min (Gain = 3600) for up to 24 h. Growth and gene expression were assessed by taking OD_600_ readings and luminescence readings, respectively. Luciferase readings were expressed as relative light units (RLU) normalised to OD_600_, to control for growth rate differences in the selected carbon sources.

**Western-blot analysis**

The cultures were grown aerobically to an OD_600_ of 0.5 in MOPS minimal medium supplemented with the indicated carbon sources. Each experiment was carried out in triplicate. The cells from 10 ml culture were sedimented at 4,000 × *g* for 10 min and then lysed in 1 × SDS solubilization buffer (Bio-Rad) at 90°C for 10 min. Equal amounts of protein were separated on a 12% (w/v) SDS—polyacrylamide gel. The proteins were blotted onto a nitrocellulose membrane, which was blocked with 5% (w/v) dried skimmed milk in PBS buffer (Oxoid™). The membranes were probed with rabbit anti-ICL, anti-MS and anti-ICD (1/5000 dilution). IRDye® 680RD and IRDye® 800CW goat anti-rabbit IgG was used as a secondary antibodies(1/10,000 dilution). Bands were visualized on an Odyssey Infrared Imaging System (LI-COR Biosciences).

Polyclonal antibodies were raised in rabbits against each of the purified proteins by Biogenes.De. The antisera were pre-absorbed against an acetone extract of a mutant strain defective in the protein of interest (e.g., the anti-ICD antisera were pre-absorbed against an acetone extract of a confirmed *icd* mutant). The cleaned-up antisera, appropriately diluted, were then used directly in Western assays.

**Biotinylated probe generation**

Biotinylated DNA probes containing the promoters of interest were PCR amplified with primers 11 and 12 (*aceA* upstream region) and 13 and 14 (*glcB* upstream region) as specified in Table S1. Sixteen 50 μl PCR reactions were pooled for each biotinylated probe (Phusion Polymerase - NEB). It was important to use large amounts of input biotinylated DNA in the subsequent immobilization procedure in order to ensure adequate saturation of the streptavidin on the beads. Unincorporated biotinylated oligonucleotides were removed using a PCR purification column (GeneJET), and the sample was concentrated to 300-400 ng/μl by ethanol precipitation. The probes were then bound to M-280 streptavidin (Dynabeads) as described previously (Jutras et al., 2012).

**Pulldown procedure**

The pulldown procedure was followed as described previously (Jutras et al., 2012) with some modifications. Briefly, 2 x 1L of *P. aeruginosa* cultures were grown in MOPS-glucose or MOPS-acetate media. The cells were harvested by centrifugation at 10,000 × *g* for 15 min at 4 °C, resuspended in lysis buffer (40 mM Tris HCl (pH 7.5), 100 mM NaCl, 10 % glycerol, 10 % sucrose, 50 mM KCl, 5 mM Cacl_2_, 10 mM HEPES). 1 x cOmplete™, mini protease inhibitor cocktail tablet (Roche) was added per 25 ml lysis buffer and cells were lysed to completion by sonication on ice. The final protein lysate (typically, 1 ml volume, 30-50 mg/ml protein concentration) was added to an aliquot (200 μl) of the DNA-coated beads and incubated with end-over-end mixing at room temperature (18-20ºC) for 30 minutes to allow binding. Salmon sperm DNA (10 μg/ml final concentration) was used as a non-specific competitor, where indicated. Following the wash steps (x3) with wash buffer (20 mM Tris HCl (pH 7.5), 10 % sucrose, 50 mM NaCl, 10 % glycerol, 10 % sucrose, 5 mM Cacl_2_, 10 mM HEPES, 50 mM KCl) bound proteins were eluted in 150 μl elution buffer (25 mM Tris HCl (pH 7.5)) containing 1 M NaCl. Samples were then desalted by acetone precipitation and the pellet was resuspended in 20 μl 1X SDS-PAGE solubilisation buffer. Samples were then analysed by SDS-PAGE (TruPAGE 4-20% gels) and stained with Coomassie Brilliant Blue.

**MS-MS Confirmation**

Mass spectrometric identification of proteins was carried out according to the strategy previously described (Hodgkinson et al., 2016). Briefly, the protein bands of interest were excised from the SDS-PAGE gel. After reduction and alkylation, proteins were digested with an excess of trypsin. Mass spectra were recorded on a Bruker UltrafleXtreme MALDI-TOF/TOF. Matrix-related ions and trypsin autolysis products were used for internal calibration. The Mascot Server was used to search the resulting peptide masses against the predicted *P. aeruginosa* PAO1 proteome.

**Supplementary Tables**

**Table S1.** Oligonucleotide primers used in the study.

| **Primer Name** | **Sequence (5’ to 3’)** | **Primer No.** |
| --- | --- | --- |
| PA5438 KO UP F | acccggggatcctctCGTACCGTCTTGTCCTGCAC | 1 |
| PA5438 KO UP R | tggttcttcatTTGGCGGGACTGGGCTATG | 2 |
| PA5438 KO DW F | cccgccaaATGAAGAACCAGGAAGAGCG | 3 |
| PA5438 KO DW R | ctgcaggtcgactctAGTCCAGCGCCAAGTACATC | 4 |
| *aceA* Tn7T lux F *BamHI* | AAACGCGGATCCCAGCGAACAGAACCAGGC | 5 |
| *aceA* Tn7T lux R *XhoI* | AAAACTCGAGGCTGCCGTTCTTCTCTTTCA | 6 |
| *glcB* Tn7T lux F *BamHI* | AAACGCGGATCCGTAGAAGTCGAGGTAGGCGG | 7 |
| *glcB* Tn7T lux R *XhoI* | AAAACTCGAGTCCAGAACGTGTCGGCAGs | 8 |
| *cco1 Tn7T lux F BamHI* | AAACGCGGATCCCCCAGCTCCAACAAACCATC | 9 |
| *cco1 Tn7T lux R XhoI* | AAAACTCGAGGACACCGAGACCCATTCCAA | 10 |
| *aceA UP F* | GGTCAATCCTTCAAGAAATGTATTGGTTG | 11 |
| *aceA R DS (Biotin tag)* | [Btn]GGTCAATCCTTCAAGAAATGTATTGGTTG | 12 |
| *glcB UP F* | CTGGTTCATCAGGGCCTC | 13 |
| *glcB R DS (Biotin tag)* | [Btn]CAATGACTGAACGCGTTCAAGT | 14 |

**Table S2:** Bacterial strains and plasmids used in this study

| **Strain or plasmid** | **Description** | **Source or references** |
| --- | --- | --- |
| **Strains** |  |  |
| *E. coli* JM109 | *E. coli* strain for cloning and expression | New England Biolabs |
| *P. aeruginosa* PAO1 | *P. aeruginosa* wild-type | Barbara Iglewski (University of Rochester, USA) |
| PAO1 *ΔPA5438* | RccR transcription factor mutant | This study |
| **Plasmids**  pEX19Gm | *P. aeruginosa* suicide vector, gentamicin resistant | (Hoang et al., 1998) |
| pUC18T-mini-Tn7T-*lux*-Gm | mini-Tn7 *luxCDABE* transcriptional fusion vector | (Choi and Schweizer, 2006) |

**Supplementary Figures**

**Figure S1** – Capture of putative regulatory proteins on immobilized DNA. The figure shows Coomassie Brilliant Blue-stained SDS-PAGE gels (4-20% gradient of polyacrylamide) of the proteins eluted from beads containing immobilized DNA segments corresponding to the upstream region of *aceA* and *glcB*. The protein samples applied to the beads came from cultures grown in MOPS minimal medium containing acetate or glucose as a sole carbon source. Protein from cultures grown in acetate as a sole carbon source are labelled with the suffix “Ac” whereas proteins from cultures grown in glucose as a sole carbon source are labelled with the suffix “Glu”. **Panel A**; pull-down carried out in the absence of added herring sperm DNA (non-specific competitor). **Panel B**; pull-down carried out in the presence of added herring sperm DNA. The molecular mass (kDa) of the markers is shown on the left hand side of each gel. The bands marked with A, B and C were selected for MS-MS analysis.


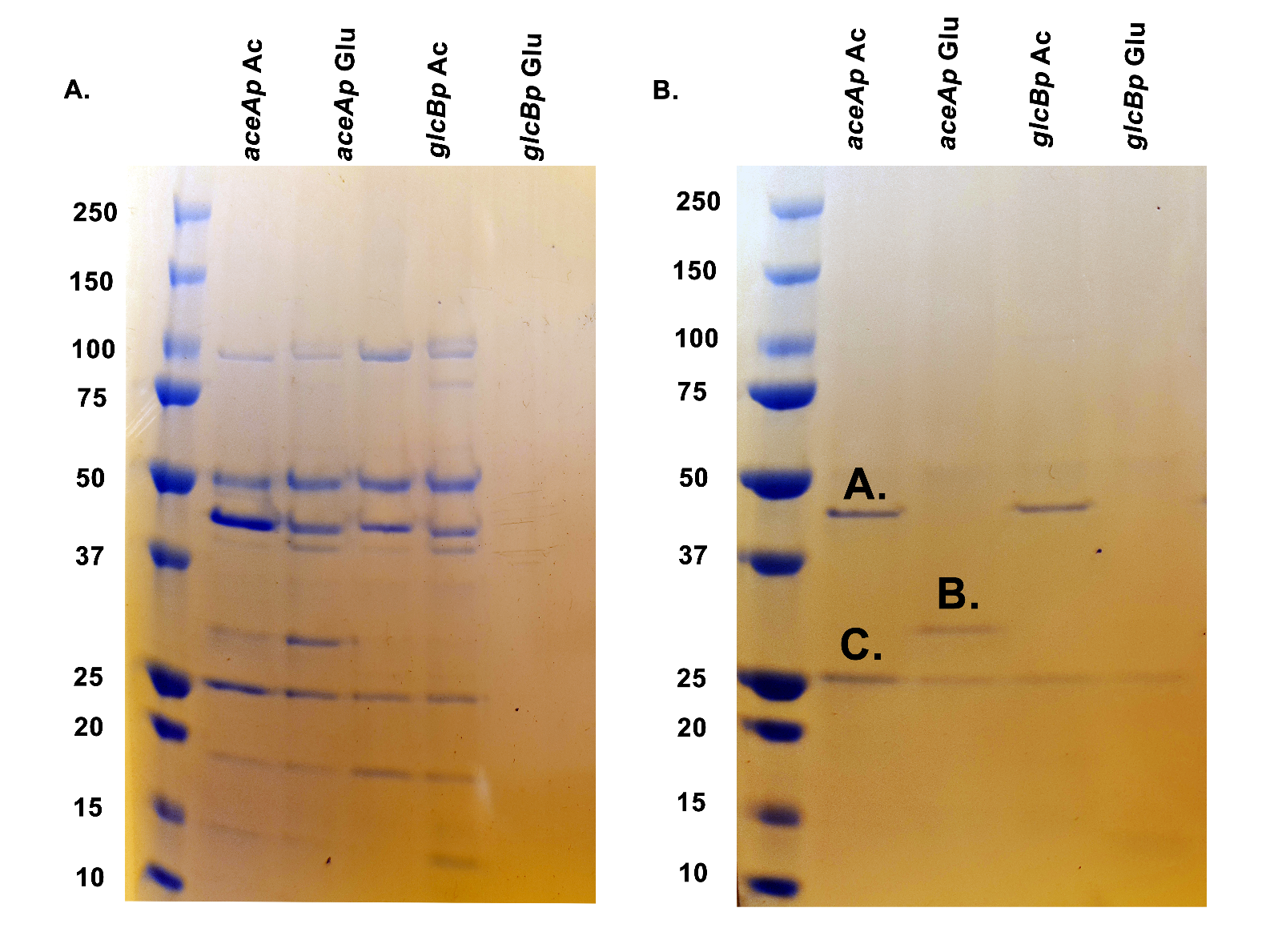


Figure S2; Mass spectrometric identification of protein bands (A-C) which bound to the *aceA*/*glcB* promoter regions.


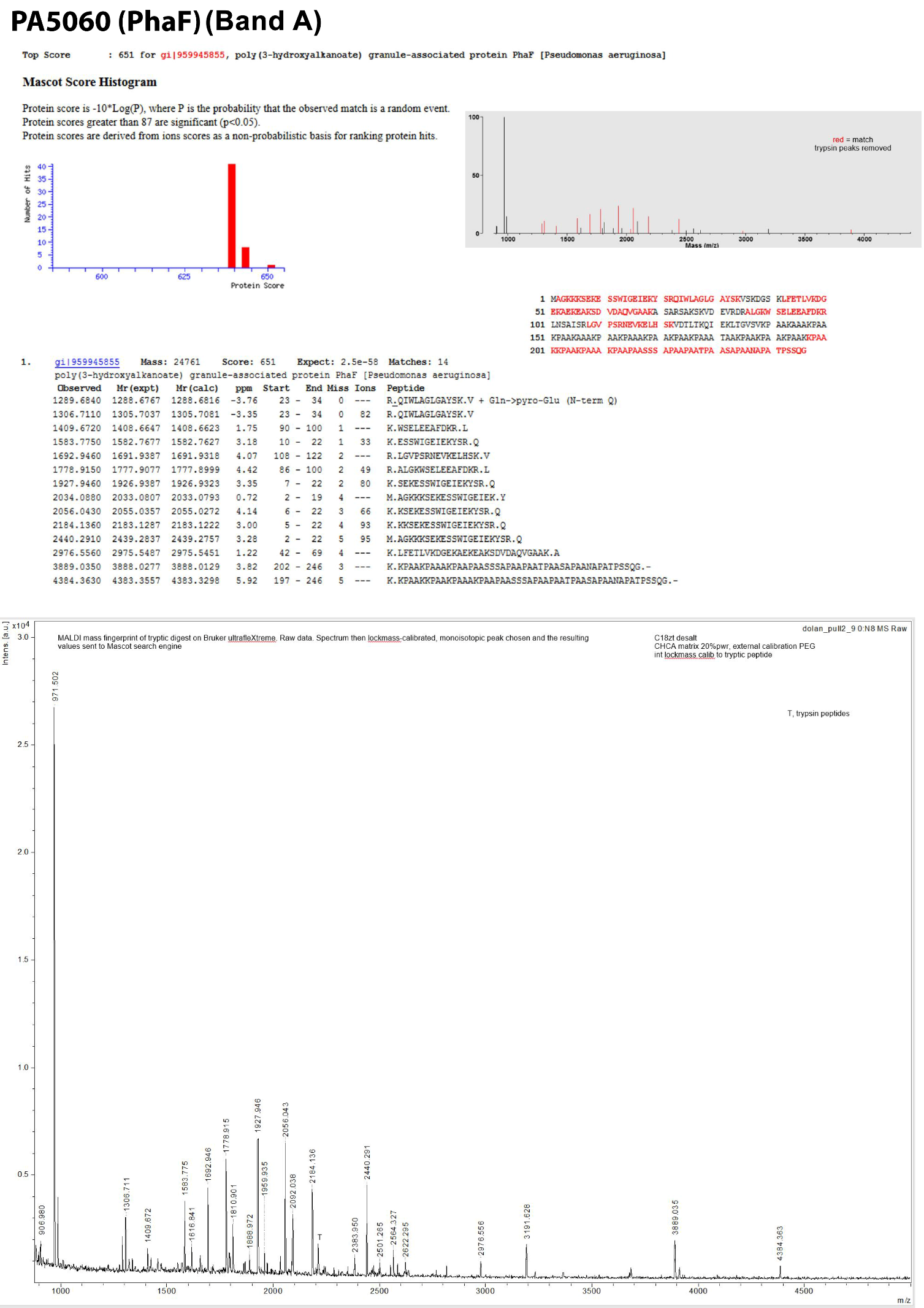


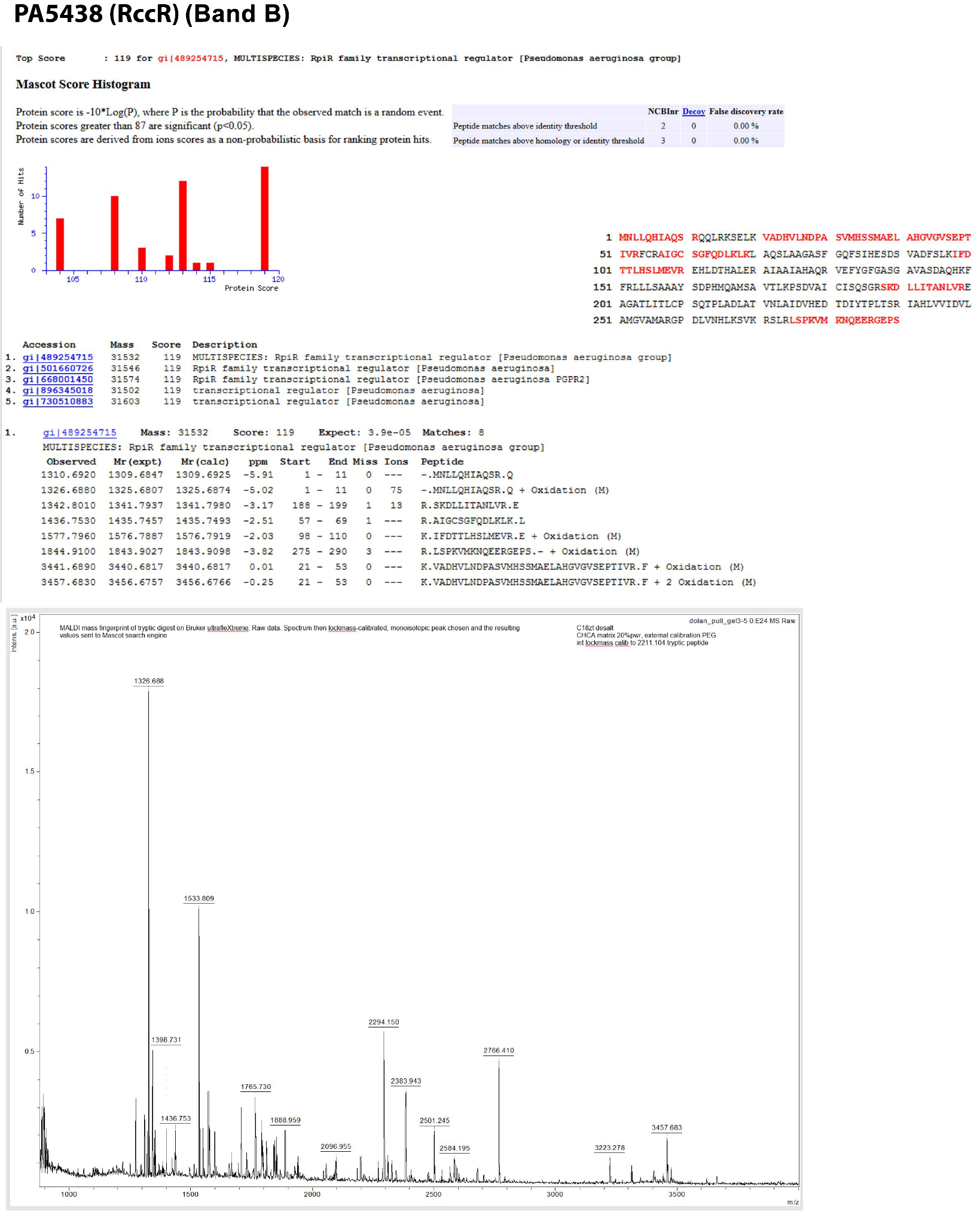


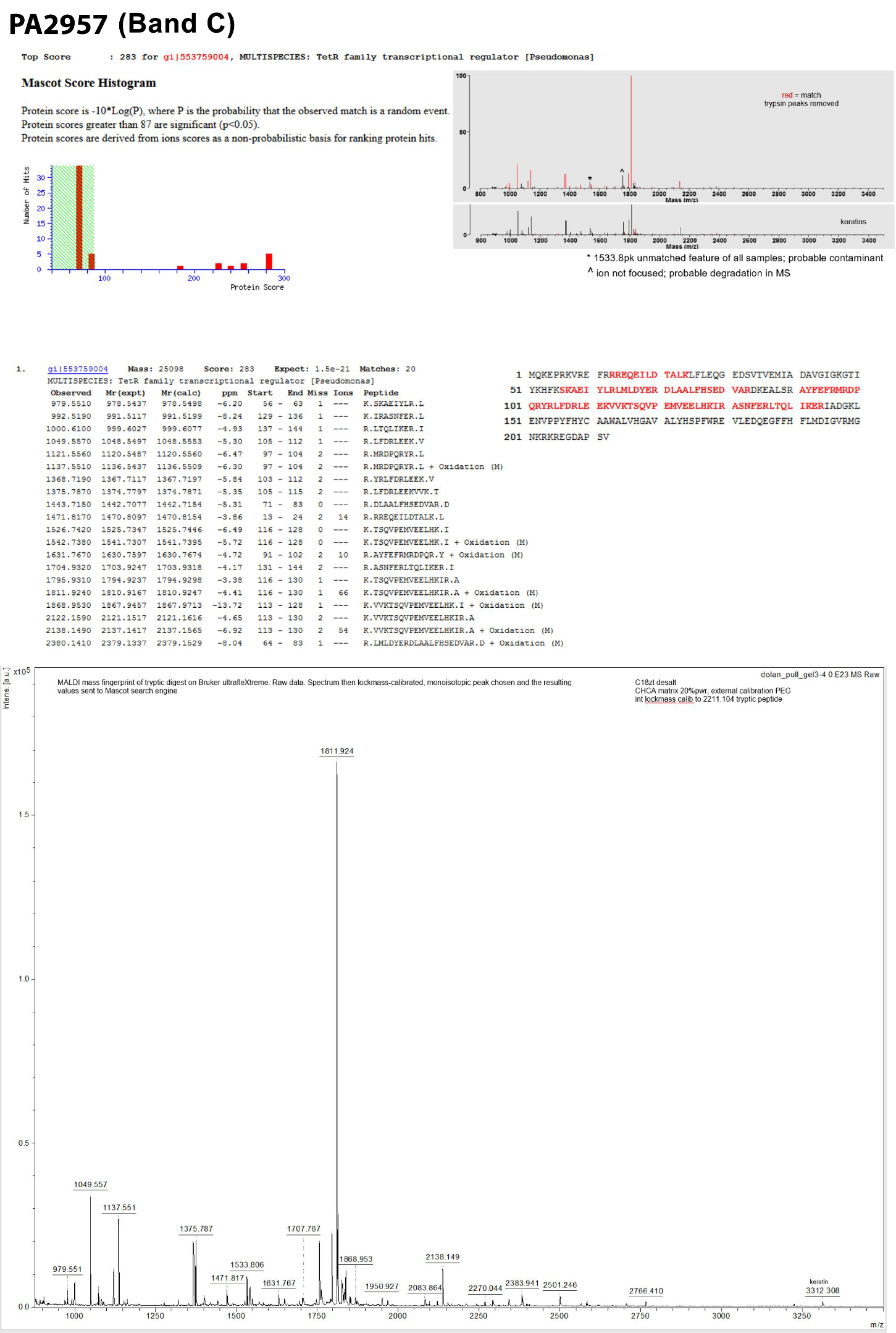


Figure S3: Growth curves of *P. aeruginosa* PAO1 wild-type (red) compared with the *ΔPA5348* mutant (blue) grown on various carbon sources. A; MOPS-Tryptone. B; MOPS-Acetate. C; MOPS-Succinate. D; MOPS-Glucose. Data represent the mean ± SD of 3 independent growth curves.


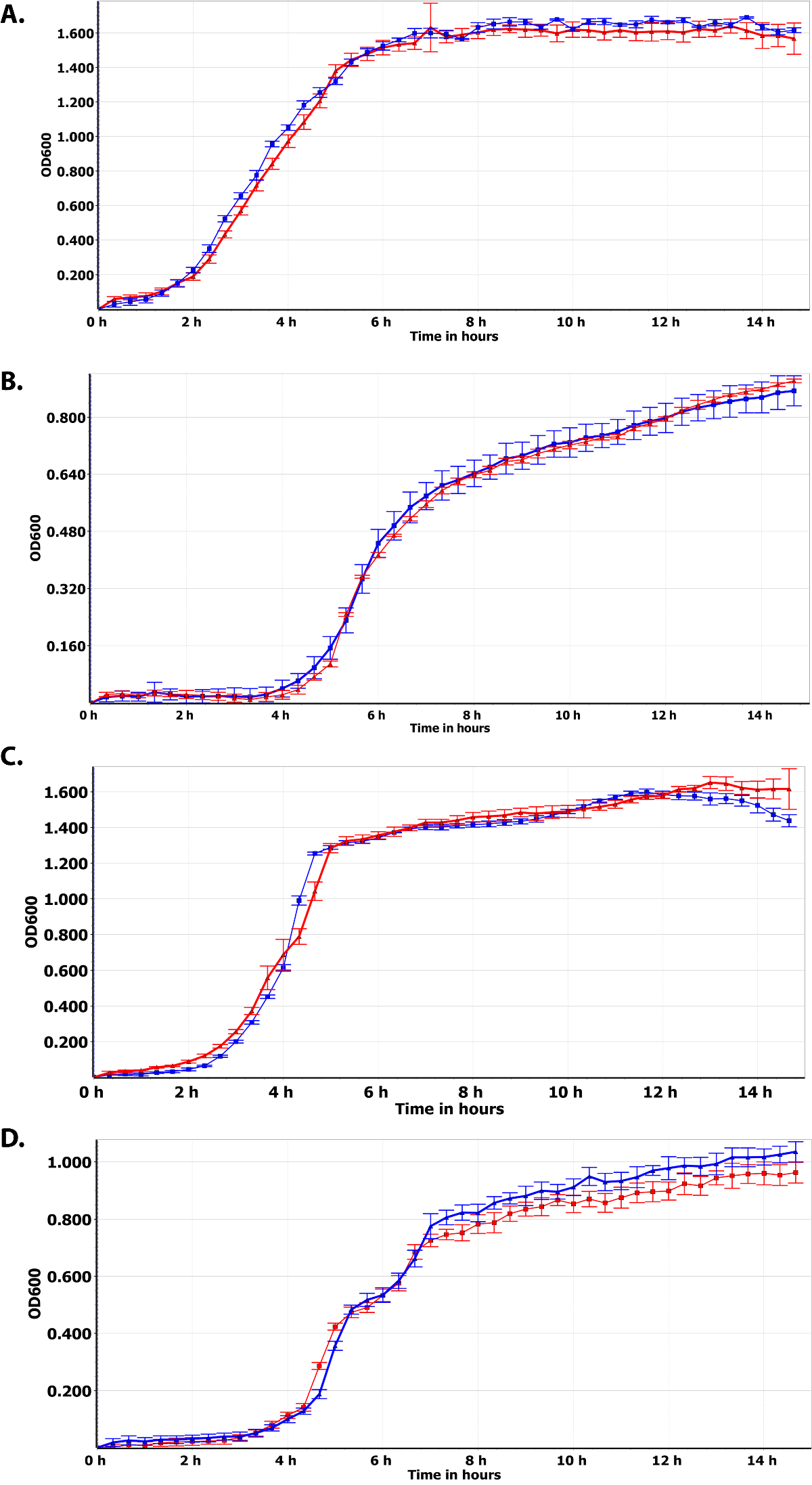


Figure S4: Quantitation of *aceA* transcriptional activity in wild-type *P. aeruginosa* (PAO1, blue line) compared with *aceA* transcription in a *ΔPA5348* mutant (red line). Transcriptional activity from the *aceA* promoter was measured using an *aceA*:lux promoter fusion, grown on various carbon sources. Panel A; MOPS Tryptone. Panel B; MOPS Acetate. Panel C; Mops Succinate. Panel D; MOPS Glucose. Lux-dependent bioluminescence is shown as relative light units (RLU).


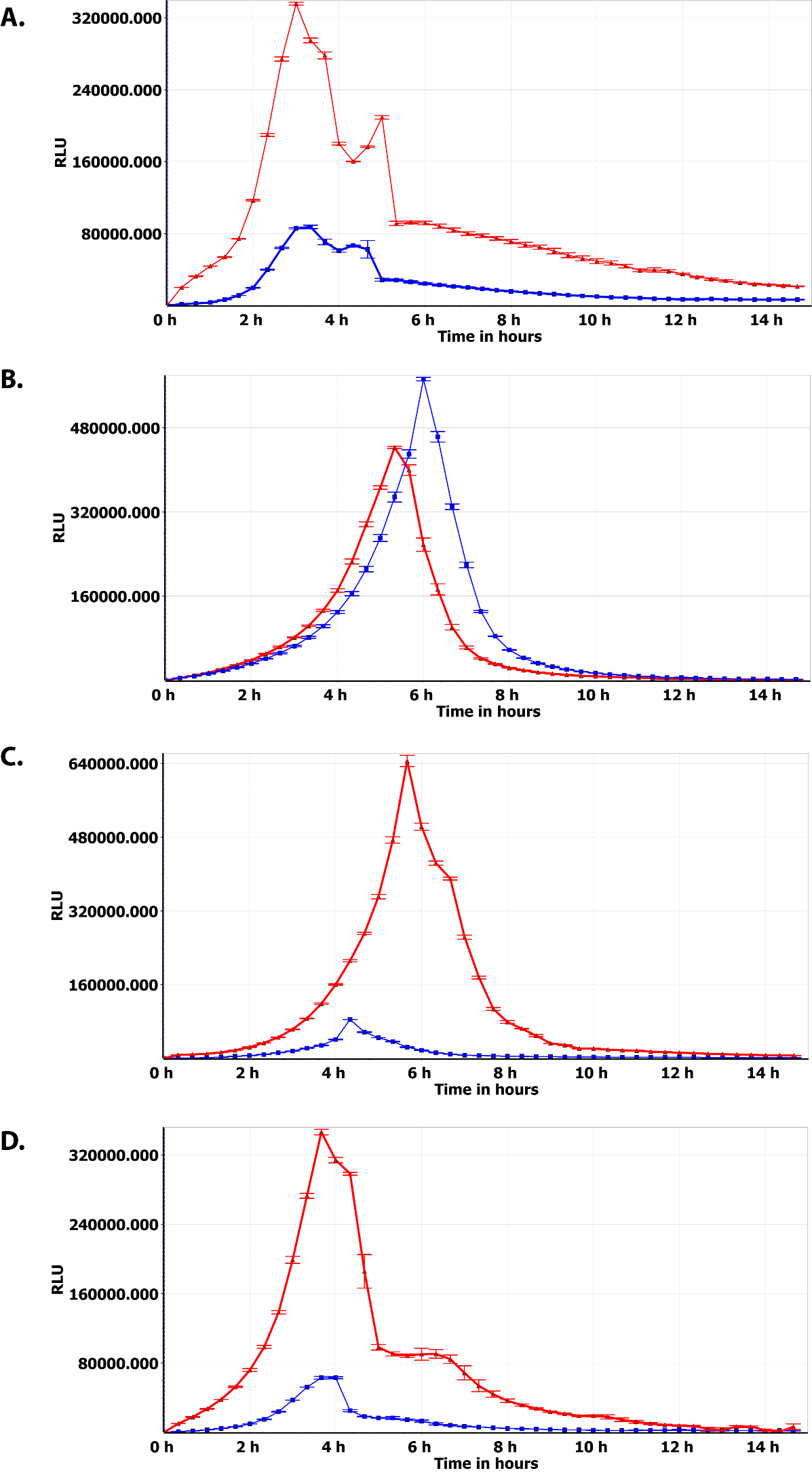


Figure S5. *glcB*:lux expression for the wild-type and the Δ*PA5438* mutant grown in MOPS minimal medium containing tryptone, acetate, glucose or succinate, as indicated. Gene expression was measured as relative light units (RLU) derived from the activity of the expressed lux enzymes. RLU values are normalised to the culture OD_600_. Data represent mean ± SD from 3 biological replicates.


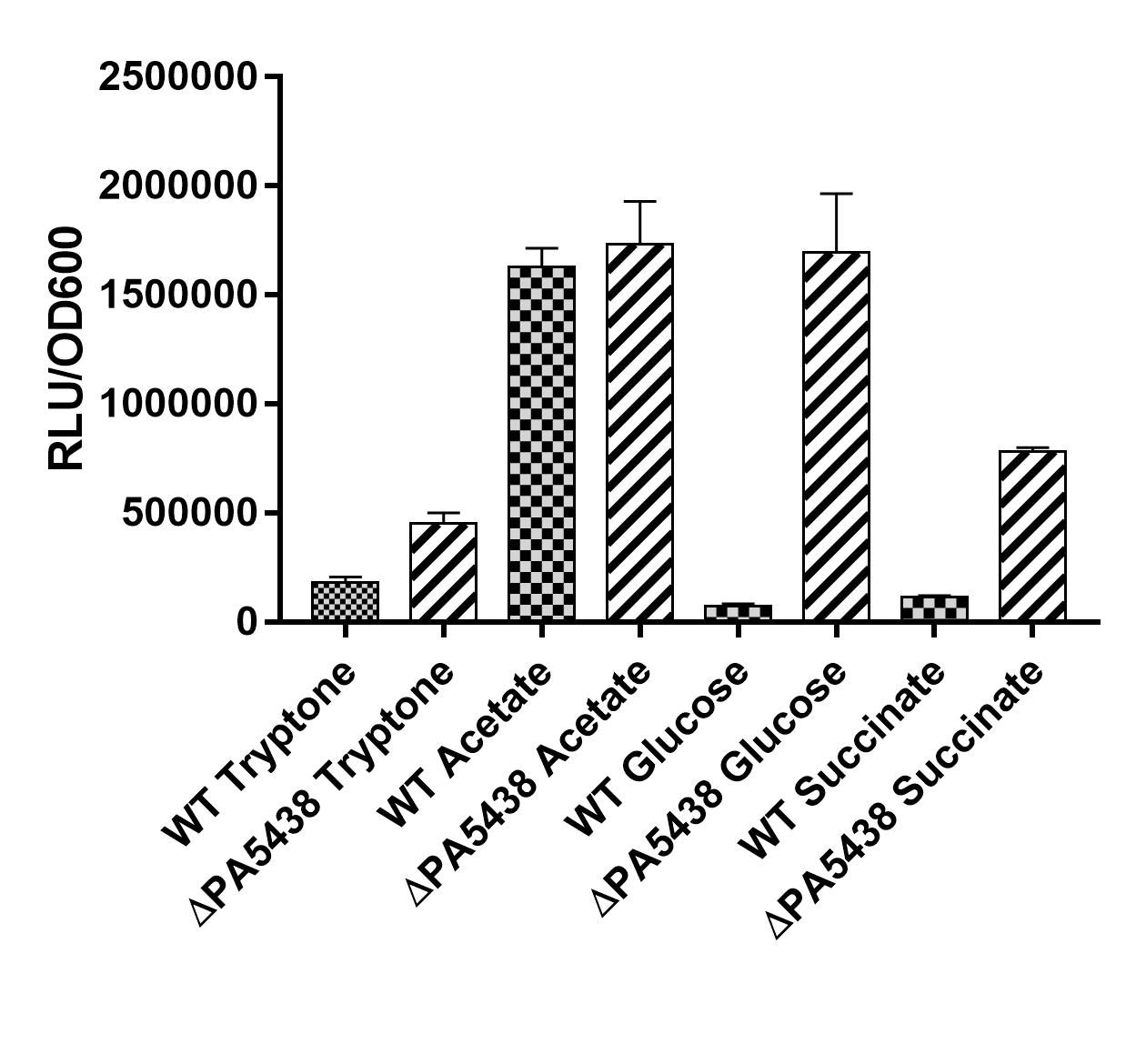


Figure S6. *cco1*:lux expression for the wild-type and the Δ*PA5438* mutant grown in MOPS minimal medium containing tryptone, acetate, glucose or succinate, as indicated. Gene expression was measured as relative light units (RLU) derived from the activity of the expressed lux enzymes. RLU values are normalised to the culture OD_600_. Data represent mean ± SD from 3 biological replicates.


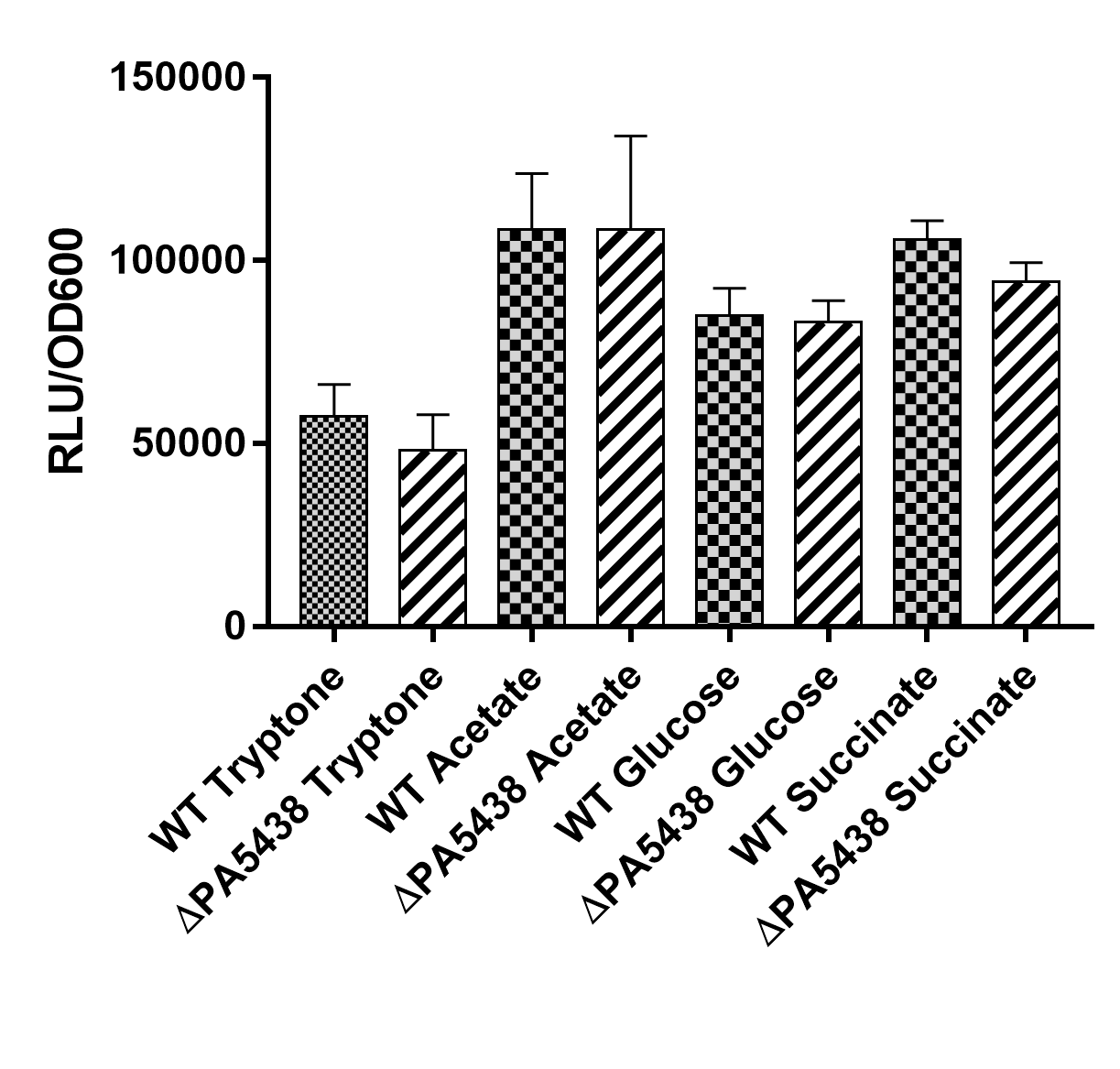


Figure S7. Western blot showing deregulated expression of AceA (59 kDa) and GlcB (79 kDa) in a *ΔPA5348* mutant compared with the wild-type during growth on the indicated carbon sources. Isocitrate dehydrogenase (ICD) was used as a loading control. Red bands indicate the molecular marker (Precision Plus Protein™ All Blue). Three replicates per sample were loaded.


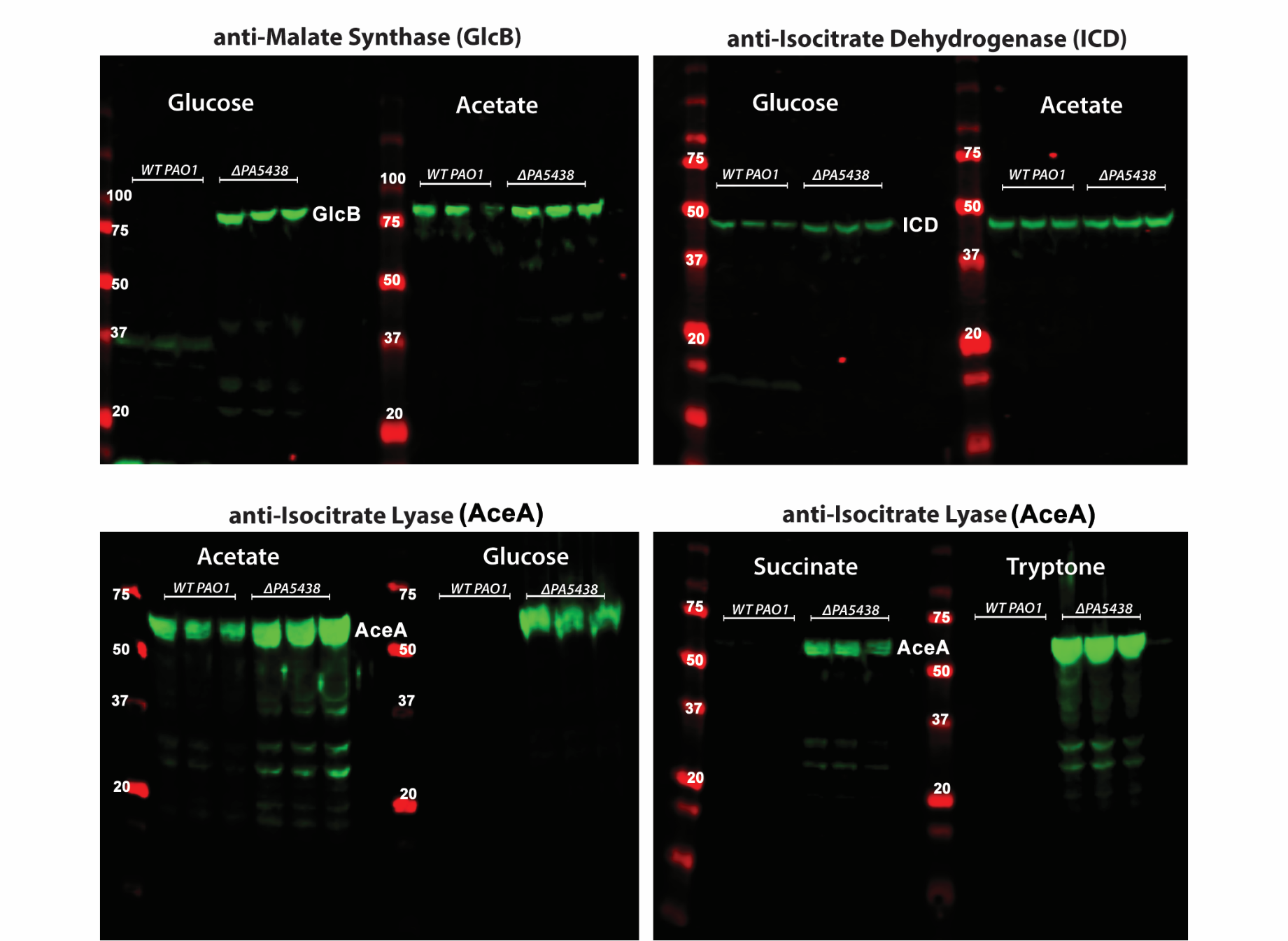


**SI References**

Choi, K.-H., and Schweizer, H.P. (2006). mini-Tn7 insertion in bacteria with single attTn7 sites: example Pseudomonas aeruginosa. Nat. Protoc. *1*, 153–161.

Heath Damron, F., McKenney, E.S., Barbier, M., Liechti, G.W., and Goldberg, J.B. (2013). Construction of Mobilizable Mini-Tn7 Vectors for Bioluminescent Detection and Single Copy Promoter lux Reporter Analysis in Gram-Negative Bacteria. CAMBRIDGE Univ. Libr.

Hoang, T.T., Karkhoff-Schweizer, R.R., Kutchma, A.J., and Schweizer, H.P. (1998). A broad-host-range Flp-FRT recombination system for site-specific excision of chromosomally-located DNA sequences: application for isolation of unmarked Pseudomonas aeruginosa mutants. Gene *212*, 77–86.

Hodgkinson, J.T., Gross, J., Baker, Y.R., Spring, D.R., and Welch, M. (2016). A new Pseudomonas quinolone signal (PQS) binding partner: MexG. Chem. Sci. *7*, 2553–2562.

Holloway, B.W., Krishnapillai, V., and Morgan, A.F. (1979). Chromosomal genetics of Pseudomonas. Microbiol. Rev. *43*, 73–102.

Holloway, B.W., Romling, U., and Tummler, B. (1994). Genomic mapping of Pseudomonas aeruginosa PAO. Microbiology *140*, 2907–2929.

Huang, W., and Wilks, A. (2017). A rapid seamless method for gene knockout in Pseudomonas aeruginosa. BMC Microbiol. *17*, 199.

Jutras, B.L., Verma, A., and Stevenson, B. (2012). Identification of novel DNA-binding proteins using DNA-affinity chromatography/pull down. Curr. Protoc. Microbiol. *Chapter 1*, Unit1F.1.

LaBauve, A.E., and Wargo, M.J. (2012). Growth and laboratory maintenance of Pseudomonas aeruginosa. Curr. Protoc. Microbiol. *Chapter 6*, Unit 6E.1.
